# Attenuation by dextromethorphan on the higher liability to morphine-induced reward, caused by prenatal exposure of morphine in rat offspring

**DOI:** 10.1186/1423-0127-16-106

**Published:** 2009-11-25

**Authors:** Ling-Yi Wu, Jain-Fang Chen, Pao-Luh Tao, Eagle Yi-Kung Huang

**Affiliations:** 1 Department of Pharmacology, National Defense Medical Center, Taipei, Taiwan; 2 Division of Endocrinology and Metabolism, Department of Internal Medicine, Tri-Service General Hospital, Nei-Hu, Taipei, Taiwan

## Abstract

Co-administration of dextromethorphan (DM) with morphine during pregnancy and throughout lactation has been found to reduce morphine physical dependence and tolerance in rat offspring. No evidence was presented, however, for the effect of DM co-administered with morphine during pregnancy on morphine-induced reward and behavioral sensitization (possibly related to the potential to induce morphine addiction) in morphine-exposed offspring. Conditioned place preference and locomotor activity tests revealed that the p60 male offspring of chronic morphine-treated female rats were more vulnerable to morphine-induced reward and behavioral sensitization. The administration of a low dose of morphine (1 mg/kg, i.p.) in these male offspring also increased the dopamine and serotonin turnover rates in the nucleus accumbens, which implied that they were more sensitive to morphine. Co-administration of DM with morphine in the dams prevented this adverse effect of morphine in the offspring rats. Thus, DM may possibly have a great potential in the prevention of higher vulnerability to psychological dependence of morphine in the offspring of morphine-addicted mothers.

## Background

Growth retardation, delayed motor development and behavior abnormalities have been proposed in offspring of heroin-addicted mothers [1]. Infants passively exposed to morphine through their addicted mothers easily developed morphine withdrawal syndrome after birth, whom even needed intensive care [2-4]. In our previous studies, we observed that many adverse effects caused by prenatal exposure of morphine could be prevented by the co-administration of dextromethorphan (DM) in morphine-dependent rat dams [5,6]. However, the possible impacts of prenatal exposure of morphine on the vulnerability to drug addiction have seldom been examined. In humans, the liability to opioid dependence can be affected by acquired physical conditions and social factors in offspring from morphine-addicted mother. Thus, we attempted to investigate the possible effects of prenatal exposure to morphine on the vulnerability to morphine-induced reward in an animal model of rats. In addition, the possible protective effect of the co-administered DM was also tested.

Being a non-competitive antagonist at the glutamatergic N-methyl-D-aspartate (NMDA) receptors, DM was thought to exert many of its pharmacological actions through the blockade of NMDA receptor [7], although DM was reported to act at the other targets (e.g. nicotinic α3β4 receptors and sigma receptors) as well [8,9]. Activation of the NMDA receptors has been implicated in the regulation of reward-related mesolimbic pathway in many reports [10-12]. Therefore, we speculated that the prenatal administration of morphine and DM to the dams may affect the development of the neural systems which will be functionally correlated with opioid-induced reward in the offspring. In order to investigate the liability to morphine-induced reward, we used conditioned place preference (CPP) test to perform quantitative verification of morphine-induced reward. The CPP tests were carried out on the rats which were the offspring from the morphine-dependent dams. The possible effect of prenatal exposure of morphine and DM on behavioral sensitization was also examined in the locomotor activity test. Using HPLC, the dopamine and the serotonin turnover rates were determined in the terminal regions of mesolimbic pathway to correlate with the results from behavioral experiments.

## Materials and methods

### Animals

Adult female Sprague-Dawley rats were purchased from the National Experimental Animal Centre, Taipei, Taiwan. The animals were housed two in a cage, in a room maintained at a temperature of 23 ± 2°C with a 12 h light-dark cycle. Food and water were available *ad libitum *throughout the experiment. Rats were randomly separated into four groups. Rats received subcutaneous (s.c.) injection of saline (control group), morphine (M group), morphine + dextromethorphan (M + DM group) and dextromethorphan (DM group) twice per day (9 AM and 5 PM) and progressively increased with 1 mg/kg at 7-day intervals from a beginning dose of 2 mg/kg for both morphine and dextromethorphan. The rats were mated on day 8 and the drug administration was continued during pregnancy. After rat offspring were born, the injections of drugs into the dams were stopped. Male offspring rats of four groups (60 days after delivery) were used for CPP test and locomotor activity test. The care of animals was carried out in accordance with institutional and international standards (Principles of Laboratory Animal Care, National Institutes of Health), and the protocol was approved by the Institutional Animal Care and Use Committee of National Defense Medical Center, Taiwan, R.O.C.

### Schedule of drug administration for CPP test and locomotor activity test

**Figure 1:**
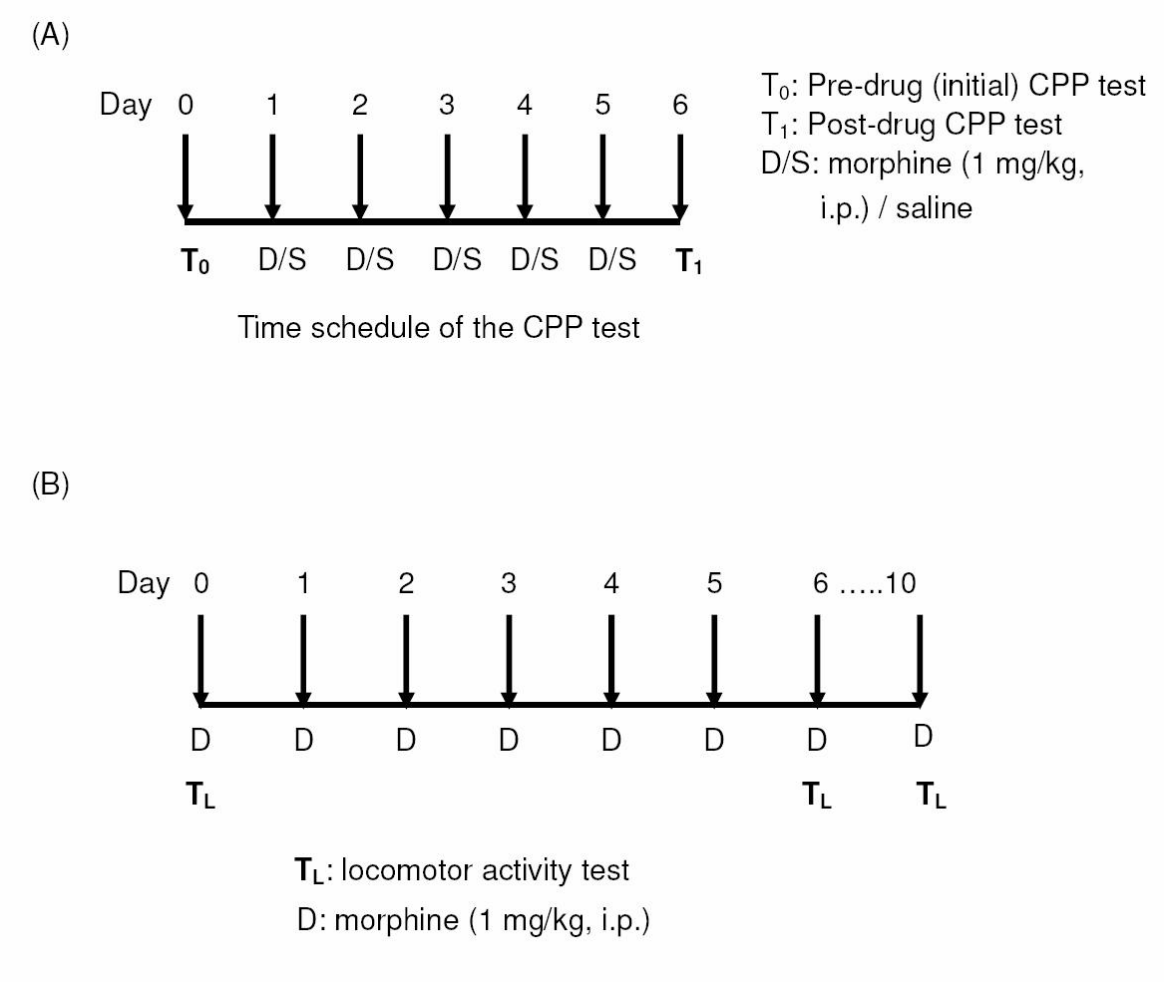
**A: Time schedule of the CPP test**. **B**: Time schedule of the locomotor activity test.

As shown in Fig. 1A, a 7-day schedule was employed for the CPP tests. One day before the start of the experiments, the animals were placed in an isolated dark room for 60 min for habituation. On day 0, pre-drug place preference was tested in the dark room. CPP conditionings and drug injections [morphine (1 mg/kg, i.p.)/saline] were performed from day 1 to day 5, alternating saline injection (i.p.) in the morning with morphine injection (i.p.) in the afternoon. Post-drug place preference was measured and recorded on day 6.

In another set of experiments, the total time of the schedule for drug administration and locomotor activity tests was 10 days (Fig. 1B). Drug administration [morphine (1 mg/kg, i.p.)] was conducted on day 0, 1, 2, 3, 4, 5, 6 and 10. One day before the start of the experiments, the animals were brought to the room and placed in the test cages for 60 min to allow them to adapt to the environment for locomotor activity test. Following injection of the drugs, the rats were immediately put into the cages for locomotor activity determination on day 0, 6, and 10.

### Conditioned place preference (CPP) test

The CPP apparatus, made from an acrylic plastic box (70 × 25 × 25 cm), was divided into three compartments. Two identically sized compartments (30 × 25 × 25 cm) were constructed at both sides, separated by a narrower compartment (10 × 25 × 25 cm). The compartments were connected by guillotine doors (10 × 10 cm) in the central unit. One of the large compartments was covered by mosaic type paper (2.5 × 2.5 cm black and white squares) on the three walls and floor, as a visual cue; the other large compartment was covered by purely white paper. To give more visual cues, a blue and a red light bulb were hung separately above the two large compartments. During the experiments, the CPP apparatus was kept in an isolated dark room, which was free from noise. After each behavioral test or place conditioning, the whole box was cleaned thoroughly to prevent interference from the smell of feces and urine. The animals were handled (30 min per day) for at least 3 days prior to the experiments. In this non-biased-design CPP apparatus, the rats were first placed into the central compartment. Their initial (pre-drug) place preference was determined by giving them free access to the entire box for 15 min. During the following 5 days, the rats were drug-conditioned for 5 times and saline-conditioned for 5 times. The animals were kept for 40 min in the corresponding compartment with the guillotine doors closed. Giving morphine in the morning and saline in the afternoon, these place conditionings were carried out to ensure the visual cue association for each rat. Post-drug place preference was examined on day 6. The test time was also 15 min.

### Locomotor activity test

The ambulatory and the total activity of rats were measured in transparent standard polypropylene animal cages (38 × 22 × 15 cm). The test cages were placed in a photo beam activity system (San Diego Instruments, U.S.A.). A computer control unit registered the interruption of photobeams from five individual cages. Ambulatory activity was recorded as breaks of two consecutive beams, whereas breaks of a single photobeam were determined as total activity. Activity was recorded in 5-min periods for 1 hour immediately after drug or saline administration. The experiments of locomotor activity were performed in an isolated noise-free room. The total and ambulatory activities recorded were both normalized as the percentage of the activity obtained on day 0 (as shown in Fig. 1B).

### High performance liquid chromatography (HPLC) analysis of dopamine and serotonin metabolites

Adult male rats (8 to 10 weeks old) from four groups were sacrificed by decapitation 30 min after a challenge of morphine injection (1 mg/kg, i.p.). The whole brain was placed in an acrylic brain matrix (1 mm section coronal, Braintree Scientific, U.S.A.). Using a sharp blade, one third of the full length of brain (without the olfactory bulbs and the cerebellum) was vertically cut off from the rostral side. A coronal slice of 1 mm was obtained by slicing at the adjacent indent of the matrix. Co-ordinating with the anterior commissure as a landmark, the neucleus accumbens (NAc) was carefully cut away from the striatum with a forceps, and the striatum was separated into dorsal and ventral portions with a horizontal cut. Following removal of the olfactory bulb, the olfactory tubercles (OT) was also carefully dissected. The medial prefrontal cortex (mPFC) was obtained from the previous slice, which was the horizontal layer above the NAc site. Tissues were homogenized in 1 mM of oxalic acid. The samples were centrifuged (4,300 g, 20 min) and filtered through an Acrodisc 25-mm syringe filter with 0.2-μm super membrane (PN: 4612, PALL Life Sciences, U.S.A.). The filtered samples were injected into the high performance liquid chromatography (HPLC) system for measurement of dopamine (DA), serotonin (5-HT), and their metabolites [dopamine metabolites: 3,4-dihydroxyphenylacetic acid (DOPAC) and homovanillic acid (HVA); serotonin metabolite: 5-hydroxyindole acetic acid (5-HIAA)]. The DA and 5-HT turnover rates were calculated to provide a better indication of their neuronal activity. The DA turnover rate was calculated as ([DOPAC] + [HVA])/[DA], while the 5-HT turnover rate was calculated as [5-HIAA]/[5-HT].

Using linear regression, standard curves were constructed, which covered a wide range of concentrations (10-1,000 nM). The samples were compared with the standard curve to determine their contents of DOPAC, HVA, DA, 5-HIAA, and 5-HT. The electrochemical chromatographic system consisted of a pump (LC-10AD, Shimadzu, Japan), a TSKgel ODS-80T_M _C18 column (Tosoh, Japan), and an electrochemical detector (Coulochem II, ESA, Chelmsford, MA, U.S.A.) containing a 5020 guard cell and 5010 analytical cells. The voltage of guard cell (mobile phase cell) was set at 350 mV. The analytical cells were set at 40 and 250 mV (detecting potential). The mobile phase (MDTM mobile phase, ESA) consisted of 75 mM sodium dehydrogenate phosphate (monohydrate), 1.7 mM 1-octanesulfonic acid (sodium salt), 100 μl/L triethylamine, 25 μM EDTA, 10% acetonitrile, pH 3.00, which was delivered at a flow rate of 1.0 ml/min.

### Statistical analysis

The data were all expressed as means ± SEM. One-way ANOVA followed by Newman-Keuls test was employed to examine the statistical significance of the difference between groups. In the tests of locomotor activity, one-way ANOVA followed by Newman-Keuls test was also used to examine the significance of the difference from the activity recorded on day 0. In the CPP tests, paired *t*-test was employed to examine the difference in the same animal before and after drug administration. *P *< 0.05 was considered statistically significant.

### Materials

Morphine hydrochloride was purchased from the National Bureau of Controlled Drugs, National Health Administration, Taipei, Taiwan, R.O.C. Dextromethorphan and all other chemicals were purchased from Sigma (St. Louis, MO, USA). The chemicals were all of analytical grade, and the solvents were of HPLC grade.

## Results

### Chronic morphine administration of the dams caused a more sensitive response to reward of morphine in the offspring rats, which could be prevented by the co-administration of DM in the dams

**Figure 2:**
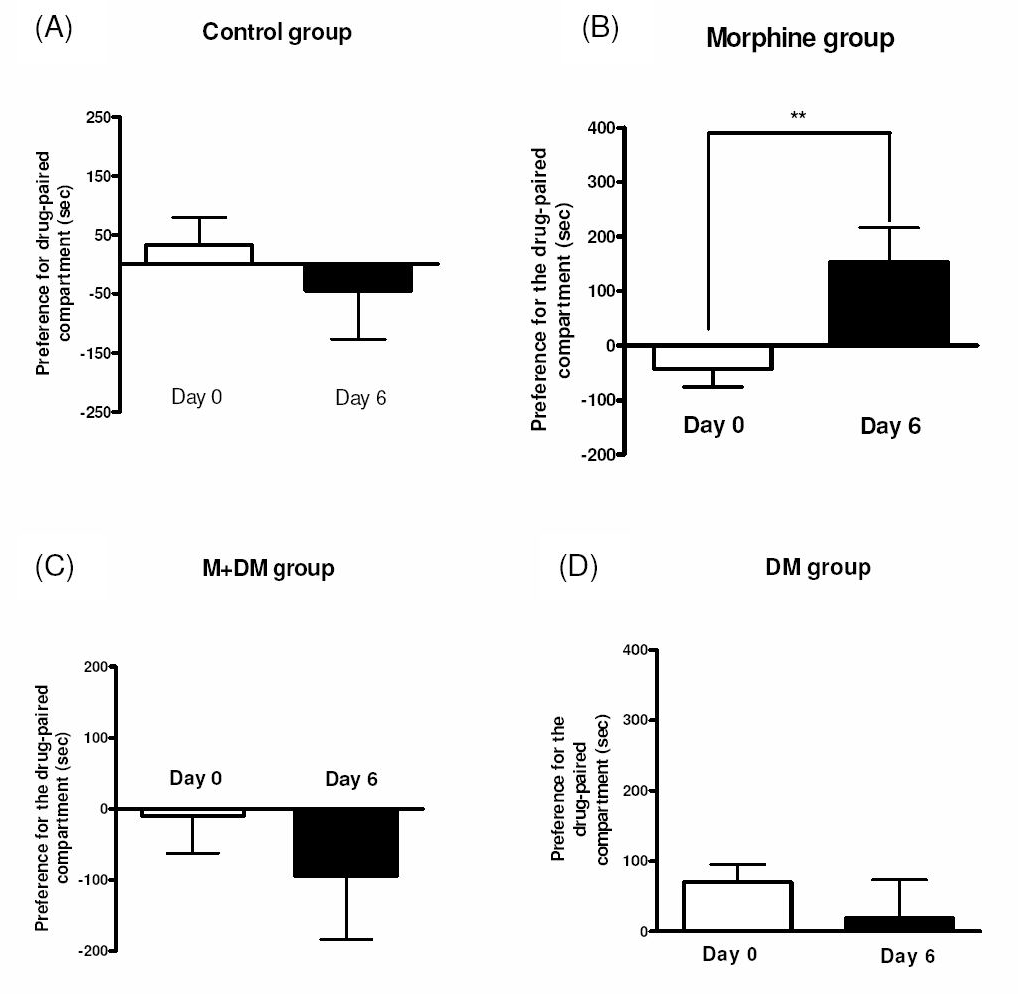
**Effect of morphine (1 mg/kg) to induce rewarding in the male offspring rats (p60) from different groups, which was measured by conditioned place preference (CPP) tests (A: Control group; B: Morphine group; C: M + DM group; D: DM group)**. Data were expressed as preference for the drug-paired compartment, which was determined by time spent in the drug-paired compartment minus time spent in the saline-paired compartment. Data were presented as mean ± SEM., and paired *t*-test was used to analyze the data (**p < 0.01 for day 6 *vs. *day 0; n ≧ 8).

As shown in Fig. 2, the time spent in the drug-associated compartment minus the time spent in the saline-associated compartment was expressed as place preference induced by drugs. Before conditioning (day 0), p60 rats of the control group showed no significant place preference (32 ± 47.6 sec) for the drug-associated compartment, which indicated that the CPP apparatus that we used was of a non-biased design [13]. After five times of low dose of morphine (1 mg/kg, i.p.) conditionings, the rats of the control group still showed no significant place preference (-44.6 ± 82.7 sec). However, rats of the morphine group showed a significant place preference for the morphine-associated compartment (from -44.5 ± 28.3 sec increased to 151.3 ± 49.3 sec) (*p *< 0.01) after conditionings. This indicated that morphine caused a significant reward in the rat offspring of the morphine group. In the DM group, the animals exhibited no place preference in response to low dose morphine conditionings (day 0: 69.8 ± 25.6 sec *vs*. day 6: 18.3 ± 55 sec). In the M + DM group, the animals also showed no place preference on both day 0 (-9.7 ± 52.8 sec) and day 6 (-93.8 ± 90 sec) (Fig. 2). This suggested that the co-administration of DM with morphine in the dams could effectively reduce the response to morphine-induced reward in the offspring rats.

### Chronic morphine administration of the dams caused a more sensitive response to morphine-induced behavioral sensitization in the offspring rats, which could be prevented by the co-administration of DM in the dams

**Figure 3:**
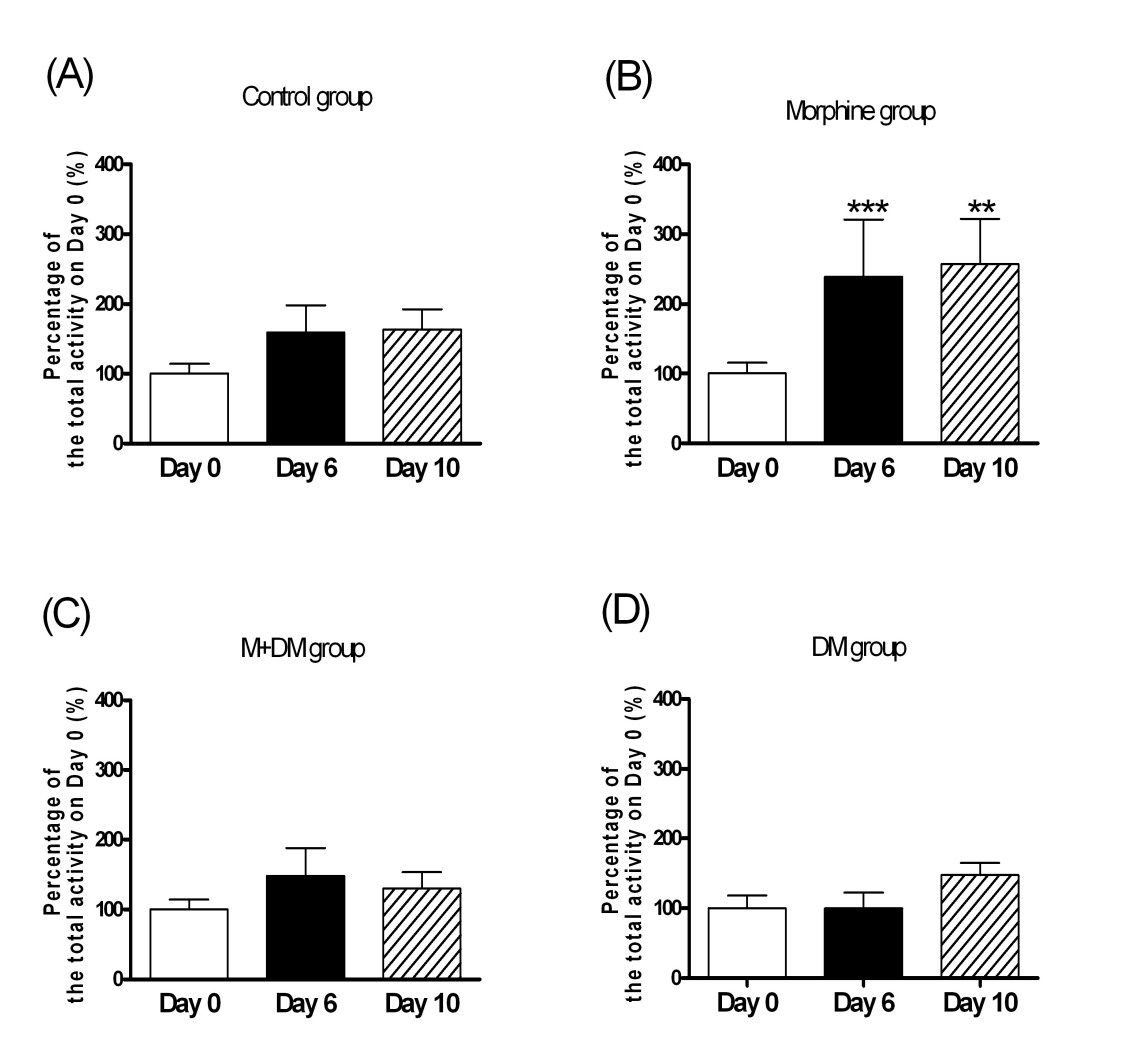
**Effects of morphine (1 mg/kg) on behavioral sensitization of total locomotor activity in the male offspring rats (p60) from different groups (A: Control group; B: Morphine group; C: M + DM group; D: DM group)**. Data were presented as mean ± SEM. Student *t*-test was used to analyze the data for the significance of the difference from the activity on day 0 (**p < 0.01, ***p < 0.001 compared with the data on day 0; n ≧ 7).

**Figure 4:**
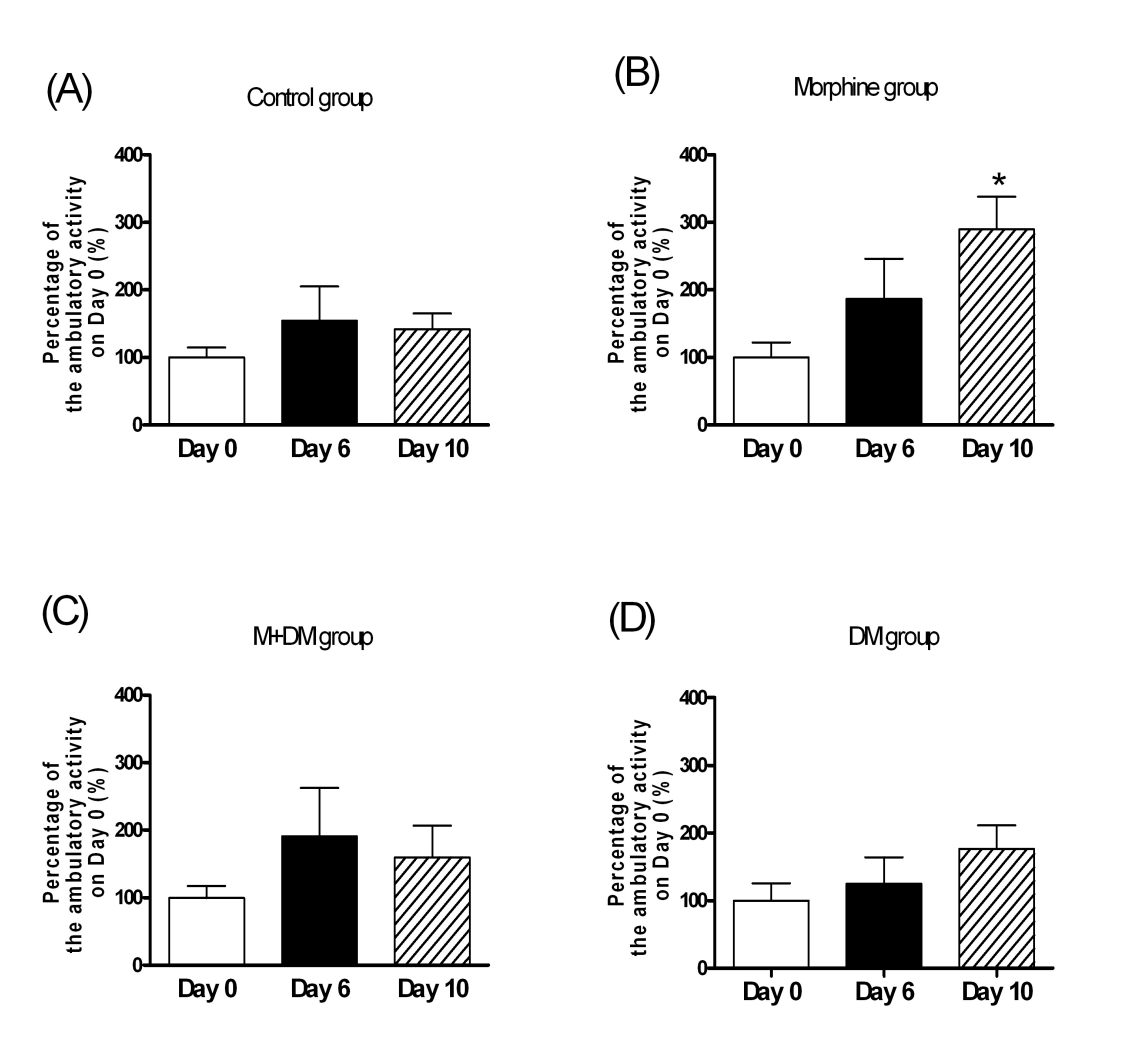
**Effects of morphine (1 mg/kg) on behavioral sensitization of ambulatory locomotor activity in the male offspring rats (p60) from different groups (A: Control group; B: Morphine group; C: M + DM group; D: DM group)**. Data were presented as mean ± SEM. Student *t*-test was used to analyze the data for the significance of the difference from the activity on day 0 (*p < 0.05 compared with the data on day 0; n ≧ 7).

For the total locomotor activity in 2 hours after injection of morphine (1 mg/kg) (Fig. 3), the rat offspring (p60) of the control group showed no significant difference among the activities recorded on day 0, day 6, and day 10. In the morphine group, the rat offspring showed a significant increase in the total locomotor activity on day 6 and day 10 when compared with the activity on day 0 after injection of morphine (1 mg/kg) (day 6: 238.6 ± 82.3% of day 0 activity, *p *< 0.001; day 10: 257.0 ± 64.8% of day 0 activity, *p *< 0.01). In the M + DM and the DM group, there was no significant difference among the activities recorded on day 0, day 6, and day 10 after injection of morphine (1 mg/kg) (Fig. 3). As shown in Fig. 4, the results of the ambulatory locomotor activity displayed a similar pattern to that of the total locomotor activity. These results indicated that chronic morphine administration of the dams caused a more sensitive response to morphine-induced behavioral sensitization in the offspring rats, which could be prevented by the co-administration of DM in the dams.

### Morphine-induced neurochemical change of dopamine and serotonin turnover rates in various brain regions obtained from the offspring of the morphine/DM/(morphine + DM)-treated dams

**Figure 5:**
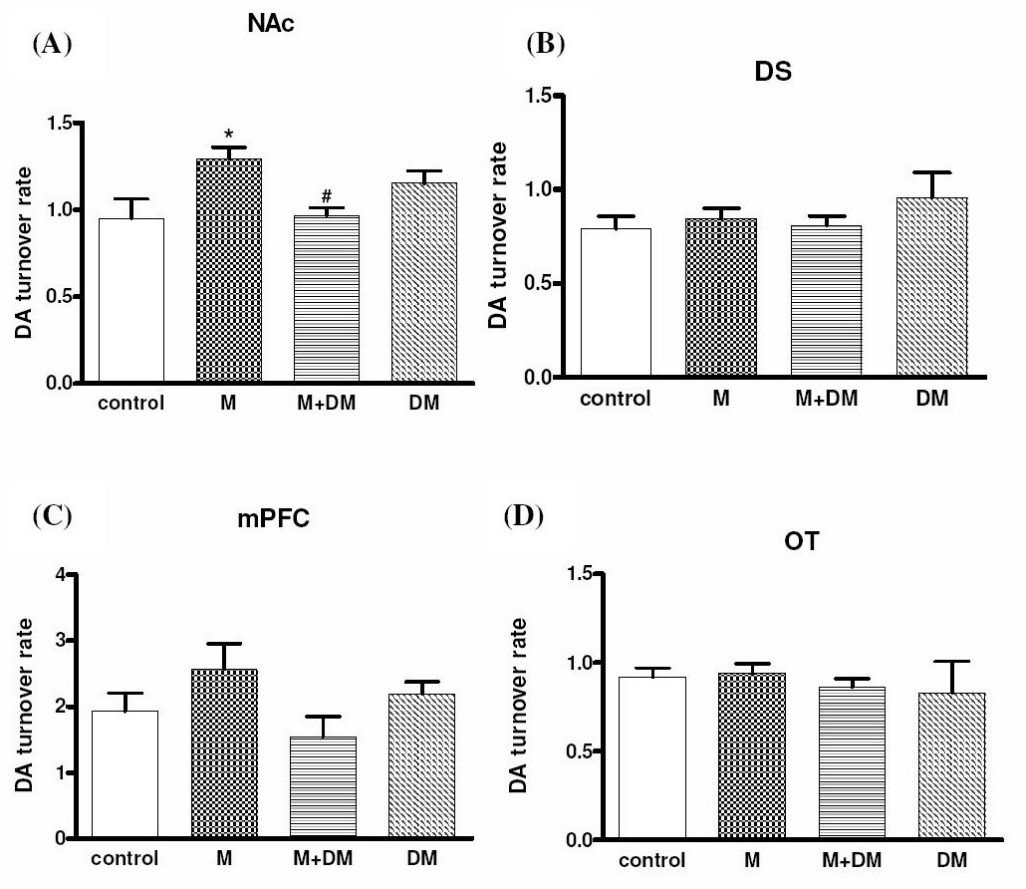
**Effects of morphine (1 mg/kg) on the dopamine turnover rate ([DOPAC+HVA]/[DA]) of the male offspring rats (p60) from different groups in the (A) nucleus accumbens (NAc), (B) dorsal striatum (DS), (C) medial prefrontal cortex (mPFC), and (D) olfactory tubercle (OT)**. Data were presented as means ± SEM. One-way ANOVA followed by Newman-Keuls test was used to analyze the data (*p < 0.05 for the morphine group *vs. *the control group; ^#^*p *< 0.05 for the M + DM group *vs. *the morphine group; n ≧ 6).

**Figure 6:**
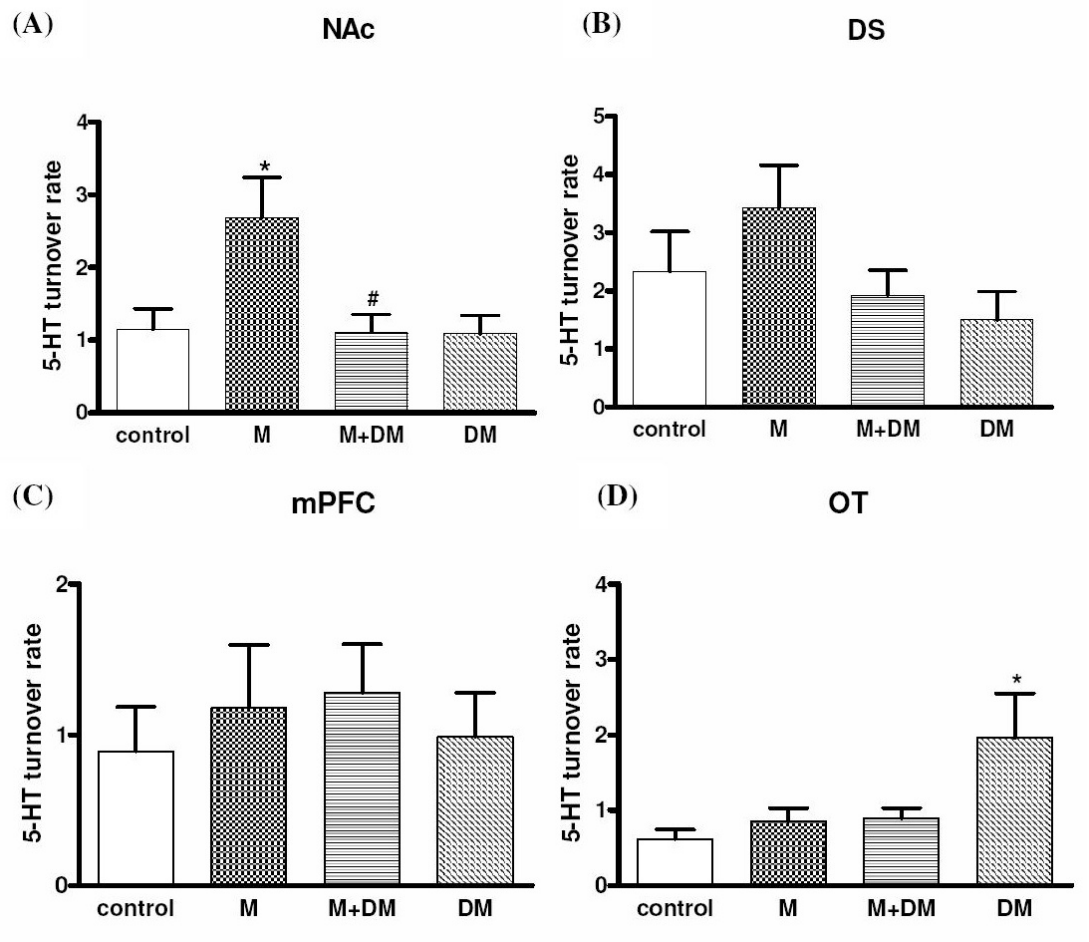
**Effects of morphine (1 mg/kg) on the dopamine turnover rate ([5-HIAA]/[5-HT]) of the male offspring rats (p60) from different groups in the (A) nucleus accumbens (NAc), (B) dorsal striatum (DS), (C) medial prefrontal cortex (mPFC), and (D) olfactory tubercle (OT)**. Data were presented as means ± SEM. One-way ANOVA followed by Newman-Keuls test was used to analyze the data (*p < 0.05 for the morphine group *vs. *the control group; ^#^*p *< 0.05 for the M + DM group *vs. *the morphine group; n ≧ 5).

As shown in Fig. 5(A), offspring rats of the morphine group showed a higher dopamine turnover rate [1.29 ± 0.06 (morphine group) *vs*. 0.95 ± 0.11 (control group); *p *< 0.05] in the NAc in response to an acute morphine challenge (1 mg/kg, i.p.). However there was no significant difference in the dorsal striatum (DS), medial prefrontal cortex (mPFC), and olfactory tubercle (OT) among different groups. In the M + DM group, the dopamine turnover rate in the NAc was not significantly different from the rates of the control group [0.96 ± 0.04 (M + DM group) *vs*. 0.95 ± 0.11 (control group)]. The dopamine turnover rate in the NAc of the animals from M + DM group was significantly lower when compared with that of the M group [0.96 ± 0.04 (M + DM group) *vs*. 1.29 ± 0.06 (morphine group); *p *< 0.05]. Consistent with the results of DA turnover rate, offspring rats of the morphine group showed a higher serotonin turnover rate [2.68 ± 0.05 (morphine group) *vs*. 1.15 ± 0.27 (control group); *p *< 0.05] in the NAc in response to an acute morphine challenge (1 mg/kg, i.p.) (Fig. 6). No significant difference was observed in the DS, PFC, and OT among different groups either. In the M + DM group, the serotonin turnover rate in the NAc was also not significantly different from the rates of the control group [1.10 ± 0.24 (M + DM group) *vs*. 1.15 ± 0.27 (control group)]. The serotonin turnover rate in the NAc of the animals from M + DM group was significantly lower when compared with that of the M group [1.10 ± 0.24 (M + DM group) *vs*. 2.68 ± 0.05 (morphine group); *p *< 0.05]. These results suggested that the mesolimbic dopaminergic system and serotoninergic system of the offspring from the dams with previous chronic morphine exposure was more sensitive to morphine, but this effect could be prevented by the co-administration of DM with morphine in the dams.

## Discussion

Previously, we found that the co-administration of dextromethorphan with morphine to dam rats throughout pregnancy significantly decreased morphine-induced physical dependence and tolerance in their offspring [5]. In correlation with morphine dependence, we investigated the possible effect of maternal morphine administration on morphine-induced reward in the CPP tests in the present study. Using a low dose of morphine (1 mg/kg, i.p.) in conditionings, the offspring from morphine-treated dams showed a significant place preference for morphine-paired compartment, but the offspring from the other groups, including control group, showed no place preference. This suggests that the offspring from morphine-addicted mother could be more sensitive to morphine-induced reward. That is to say, they could be more vulnerable to morphine dependence and easier to become addicted to morphine after first postnatal morphine exposure. Maternal co-administration of DM with morphine also abolished the effect of morphine on the CPP in the offspring. In 1997, Gagin et al. first demonstrated that prenatal morphine exposure (gestational days 12-18) enhances morphine-induced CPP in adult rats [14]. Our results were consistent with the finding by Gagin et al., but the morphine administration was throughout the pregnancy in our protocol. A recent report by Riley and Vathy showed that mid- to late gestational morphine exposure did not alter the rewarding properties of morphine in adult male rats [15]. In this study, they used many doses of morphine in conditionings of their CPP paradigm. Because the dose-dependent CPP of morphine was not observed, they interpreted their data as no effect of prenatal morphine exposure. In fact, only the offspring from morphine-treated mother showed a possibly significant CPP by morphine after low dose (0.1 mg/kg, s.c.) of morphine conditionings in their study, whereas the other groups of rats did not respond to this dose of morphine. To compare with our results, the controversial different conclusions between ours and the one from Riley and Vathy could be due to the sensitivity of CPP paradigm and the selection of marginal doses of morphine to induce CPP.

In order to provide the neurochemical evidence, we investigated the change of dopamine and serotonin turnover at the terminals of mesocorticolimbic pathway in response to low dose (1 mg/kg) of morphine. The present results showed that mesocorticolimbic system of rat offspring of morphine group was more sensitive to morphine, but the rat offspring received the co-administration of DM with morphine did not show this effect. Interestingly, the elevated dopamine turnover rate caused by prenatal morphine was only observed at the NAc, but not mPFC. Since we used a marginal low dose of morphine to induce reward, the mesolimbic pathway, originating from VTA and terminating at NAc, could be more sensitive in response to morphine, when compared with the mesocortical pathway. Although the mechanism of DM to reduce the sensitivity of mesocorticolimbic system was still unknown, these neurochemical data supported the finding of behavioral experiments and further highlighted the possible therapeutic potential of DM. In addition to CPP, we examined another behavioral parameter, behavioral sensitization of locomotor activity, which has been viewed as an important factor in the development and maintenance of addiction. Repeated administration of morphine results in augmentation of locomotor and/or stereotype behaviors [16-18]. The phenomenon could persist for a long period after morphine withdrawal and was called as "behavioral sensitization" [19]. This behavior was also presumed to be caused by the sensitization of mesolimbic dopaminergic pathway [20,21]. Our results of behavioral sensitization showed exactly the same pattern as those tested in the CPP experiments. This reconfirmed the effect of prenatal morphine exposure to cause a more sensitized mesolimbic pathway, and the effect of co-administered DM to decrease the prenatal impact of morphine in the offspring.

In our neurochemical results of 5-HT, we found that the serotoninergic projections to NAc were also more sensitive to morphine stimulation to release 5-HT in offspring from morphine-treated dams. Although the role of 5-HT was traditionally thought to be one of the neuro-substrates in producing reward, a recent report by Weitemier and Murphy confirmed that serotoninergic and dopaminergic systems at the NAc play modulatory roles in concert for morphine-induced place preference [22]. Interestingly, the only brain region where we observed significant differences for 5-HT and DA turnover was both at the NAc. Although the mPFC and OT were also known as the terminals of mesolimbocortical pathways, the NAc was most relevant and may directly link to the induction of reward. The data at the DS was found to be with no difference between groups, which may serve as the negative control to verify our neurochemical analysis. Anyhow, the release of 5-HT at NAc was more sensitive in response to morphine in the offspring from morphine-treated dams, which may also contribute to the higher liability to morphine-induced reward.

Regarding to the mechanism of DM to reduce the adverse effects of prenatal morphine, many behavioral studies revealed that NMDA receptors are involved in the development of rewarding effect related to psychological dependence caused by chronic morphine exposure [23,24]. In our previous study, we also showed that the co-administration of ifenprodil (a selective NR2B subunit-containing NMDA receptor antagonist) with morphine at the NAc, not at the VTA, could reduce morphine rewarding and drug-seeking effect, but not behavioral sensitization [25]. Another report demonstrated that the NMDA receptor antagonists: dizocilpine and ketamine, which have similar affinity for NR2A and NR2B subunit-containing NMDA receptors, suppress morphine-induced rewarding effect [26]. These results suggest that the NR2B subunit-containing NMDA receptor in the NAc may be associated with the rewarding effect of morphine. Therefore, DM may possibly act through the blockade of NMDA receptors to affect morphine-induced reward in offspring from morphine-treated dams, although there was no direct evidence showing the link between the prenatal blockade of NMDA receptors and the increased sensitivity of mesolimbic dopaminergic pathway in the literatures. Since the blockade of NMDA receptors could be able to affect numbers of various protein expressions during prenatal stage, the possibility of increased or decreased functional proteins (e.g. receptors, transporters etc.) should be taken into account for the cause of sensitized mesolimbic system in adulthood. During development, the neural change could be complicated and the relevant mechanisms of prenatal DM remain to be tested in further investigations.

In summary, the present study provides behavioral and neurochemical evidences in neonatal rats passively exposed to morphine throughout embryo stages, which suggest that they could be easier to develop many adverse effects, possibly including psychological dependence of morphine. Therapeutically, DM could reverse these adverse effects caused by prenatal morphine. The current results implied the possible biological change in offspring from morphine-addicted mother in humans. Moreover, the therapeutic potential of DM was further highlighted; especially our previous report also indicated the ability of DM to reduce morphine-induced hyperprolactinemia in female rats at different reproductive stages [27].

## Competing interests

The authors declare that they have no competing interests.

## Authors' contributions

LYW and JFC carried out the experiments. PLT and EYH conceived of the study, and participated in its design and coordination. All authors read and approved the final manuscript.
